# Anterior Incision Offloading for Primary and Revision Total Ankle Replacement: A Comparative Analysis of Two Techniques

**DOI:** 10.2174/1874325001711010678

**Published:** 2017-07-31

**Authors:** Andrew D. Elliott, Thomas S. Roukis

**Affiliations:** Gundersen Health System, Orthopaedic Center, La Crosse, WI, USA

**Keywords:** Anterior Incision Site, Delayed Wound Healing, Offloading, Post-Operative Complication, Sir Robert Jones Compression Dressing, Total Ankle Arthroplasty

## Abstract

**Background:**

There exists a high risk of post-operative complications with primary and revision total ankle replacement surgery. Delayed wound healing of the anterior incision is common. The reason for this is multi-factorial and, to date, most of the research has focused on predisposing factors involving the patients themselves. Only recently have researchers begun to look at the post-operative dressing as a possible consideration when trying to prevent incision wound healing complications. Currently, no standard post-operative dressing for primary or revision total ankle replacement exists. However, the principles of post-operative edema reduction to improve healing, as advocated by Sir Robert Jones and demonstrated in his compressive dressing, have been known for decades. We have been using a modified Sir Robert Jones compressive dressing for both primary and revision total ankle replacements. Recently, we have added an aperture pad made of cotton cast padding over the anterior incision in order to protect the area from pressure necrosis.

**Methods:**

This is a comparison study of the post-operative wound complications involving 35 patients that received the original dressing and 33 patients that received the addition of the aperture pad.

**Results:**

With no significant difference in the patient populations, the results demonstrate a 3-fold decrease in the number of anterior incision wound healing complications with the use of the aperture pad.

**Conclusion:**

This dressing represents a simple, reproducible, easy to apply and inexpensive way to prevent post-operative edema and anterior incision wound healing complications.

## 1 INTRODUCTION

For end stage ankle arthritis there remain two primary options for treatment: ankle arthrodesis and total ankle replacement. Ankle arthrodesis has long been the preferred and more predictable method of treatment; however, as total ankle replacement emerges from its infancy, it has come to rival ankle arthrodesis as a viable treatment alternative [1]. Even with advances in surgical technique and prosthesis design, total ankle replacement is not without its risks. Glazebrook *et al.* [2] reviewed over 2,000 total ankle replacements and reported an overall failure rate of 12.4% at 5.3-years. In this group, incision wound healing problems were the 4^th^ most commonly reported complication. Raikin *et al.* [3] looked specifically at patient risk factors for incision healing and found that of 106 total ankle replacements studied only 70 (66%) healed uneventfully. The majority of total ankle replacements require the use of an anterior ankle incision for implantation. While this approach provides excellent exposure, it is also problematic. Looking globally, the reasons for the incision wound healing complications can be divided into three areas: the lower extremity anatomy/angiosomes involved, patient risk factors and surgeon risk factors.

Taylor and Palmer famously described the angiosome principle as a three dimensional block of tissue fed by source arteries [4]. Attinger and colleagues [5] focused on this work involving a detailed examination of blood supply to the foot and ankle. They noted that the foot is an end organ with 6 angiosomes that, through direct arterial-arterial connections or indirect choke vessels, are capable of supplying blood to one another. When planning surgical incisions in a patient with normal blood flow, they advise making the incision along the border between two adjacent angiosomes [5]. The anterior ankle incision for a total ankle replacement is made, not in this preferred border area, but to the central aspect of a single angiosome fed by the anterior tibial artery. Due to the position of the incision, violations of this artery or associated perforating arteries will be made as far away as possible from the assistance of choke vessels; thereby minimizing the effect of the collateral circulation present in the foot. Anatomically, the deep peroneal nerve and anterior tibial artery both are superficial and are located very near the incision itself. This neurovascular bundle must be correctly identified and safely mobilized for appropriate exposure [6]. This technique places the neurovascular bundle at risk for compromise both during the initial dissection and mobilization process. The soft-tissue around the ankle also poses a problem in that it is a thin envelope and does not readily allow for the more robust soft-tissue coverage seen in knee and hip arthroplasties. Without the added protection, the area is placed at a heighted risk for wounding [7].

Patient risk factors are those inherent in the patient themselves. Careful patient selection is important when contemplating total ankle replacement. The majority of studies have examined the effect of the patient on the prosthesis itself. Fewer studies have looked at the effect of the patient on incision wound healing. In a 2010 study, after noting an incidence of wound healing complication of 28%, Whalen *et al.* [8] retrospectively examined 57 primary total ankle replacements performed by them. They concluded that there was a statistically significant increase in rate of incision wound breakdown associated with smoking greater than 12-pack-years regardless of stop date, peripheral vascular disease and cardiovascular disease [8]. In the previously mentioned study by Rankin *et al.* [3], the patient outcomes were divided into three categories: no complications; minor complications defined as those that resolved with local wound care or oral antibiotics; and major complication defined as those requiring a return to the operating room. When comparing these three patient populations, Rankin *et al.* [3] concluded that the only condition that was significant between those with no complication and those with minor complications was a diagnosis of diabetes mellitus. When they combined those with no complications with those with minor complications and compared them with those with major complications, it showed that the significant factors were women with a history of inflammatory arthritis coupled with a history of corticosteroid use [3]. This is the prototypical rheumatoid patient who tends to present with vascular stiffness or fragility.

Lastly, there are those risk factors involved with the surgeon and their surgical acumen. During the procedure, the surgeon should: limit soft-tissue dissection and periosteal stripping from the distal tibia and talus; practice meticulous hemostasis to avoid hematoma; handle tissues gently; and perform a layered closure. Violations of any of these principles result in the risk of incision wound complications. Once the incision is closed other problems present themselves. The anterior ankle is a technically challenging area of the body to apply a dressing. The cast padding tends to bunch and constrict in that area, creating a risk for pressure wounds. In a study by Lee *et al.* [9] looking at iatrogenically created wounds, they found that splint and dressing induced ulcers were the second leading cause of referral to their plastic surgery department for extremity reconstruction.

However, the primary enemy of skin healing is post-operative edema. Edema compromises local circulation and increases the potential for skin necrosis which then leads to incision dehiscence and delayed incision healing. In an attempt to address these concerns, Matsumoto and Parekh [10] published a 2015 study on the use of negative pressure wound therapy (NPWT) applied to the anterior incision site at the time of total ankle replacement surgery. They noted an incidence of anterior incision wound healing problems to be as high as 34%. Seeing a problem, the authors performed a retrospective cohort study comparing those that had NPWT applied to their anterior ankle incisions immediately upon closure and those that did not. The single-use disposable NPWT was set at 100-mmHg and remained in place for one week after surgery. The groups were evenly divided with 37 participants in each and with no significant difference in the demographics or co-morbidities. The results were 9 (24%) incision wound healing problems in the control group and 1 (3%) in the NPWT group. NPWT was found to reduce wound healing problems with an odds ratio of 0.10 (95% CI, 0.01-0.50; p = .014). The cost of the NPWT was estimated in the United States to be $220 with no additional needs for dressing changes. In 2014, Hsu *et al.* [11] in a “Technique Tip” article attempted to address the incision healing problems with a multilayered compression dressing that decreased the pressure directly over the anterior total ankle replacement operative wound by reducing surrounding tissue edema. This dressing was changed by a physical therapist 2 to 3 times per week until the sutures were removed at approximately 2.5-weeks. The data provided for patient outcomes was limited. They did report that for 100 patients only 2 had major wound complications during their undefined study period. The authors did not report on minor wound complications, mean duration of indwelling closed-suction drain use, mean time until suture removal nor was the actual cost of the dressing technique reported. The authors did state that this was an inexpensive dressing. However, the use of a skilled technician to perform the 2 to 3 times weekly dressing changes until the site is healed may pose a significant expense for the clinician and an inconveniently large time commitment by the patient.

**Fig. (1):**
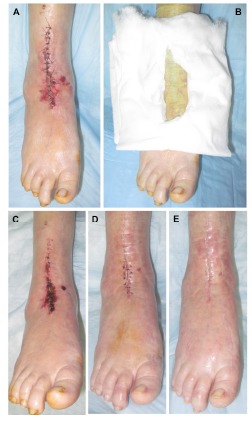
Photographic montage of the alpha patient for which the anterior offloading aperture pad was created. Initial appearance at the time of dressing change 7-days post-operative demonstrating pressure necrosis and surrounding incisional unstageable deep tissue injury (A). Completed anterior offloading aperture pad with additional medial cut-out to reduce pressure throughout the region of pressure necrosis (B). Note that a non-adherent contact layer dressing has been applied between the skin and aperture dressing. Subsequent appearance 4-weeks post-operative demonstrating stabilization of the anterior ankle incision and extent of dry eschar (C) that underwent excision and layered closure in the office setting at this time (D). Well-healed anterior ankle incision demonstrated at 8-weeks post-operative (E).

With proper patient selection, knowledgeable dissection and careful tissue handling some of the risk factors for anterior incision wound complications can be mitigated. However, the importance of the dressing cannot be overlooked in its role in reducing those same complications. We encountered a patient who developed anterior incision wound healing following revision total ankle replacement directly attributable to pressure from the dressing. During their subsequent care we recognized this and created an aperture within folded cotton cast padding to purposefully offload the anterior ankle incision. The wound stabilized almost immediately, ultimately underwent excision and layered closure, and proceeded to full healing without further complications (Fig. **1**). Based on this patient’s response to the cotton aperture offloading padding, we subsequently applied this approach to all patients undergoing primary and revision total ankle replacement. With limited published data available to guide dressing selection, we elected to evaluate our current dressing practice and compare the modified Sir Robert Jones dressing alone with the modified Sir Robert Jones dressing incorporating the cotton aperture offloading dressing technique. Accordingly, we present a retrospective comparative study comparing these two dressing techniques to determine if there was any reduction in anterior ankle incision site wound healing during the period of immobilization.

## 2 PATIENTS AND METHODS

**Fig. (2):**
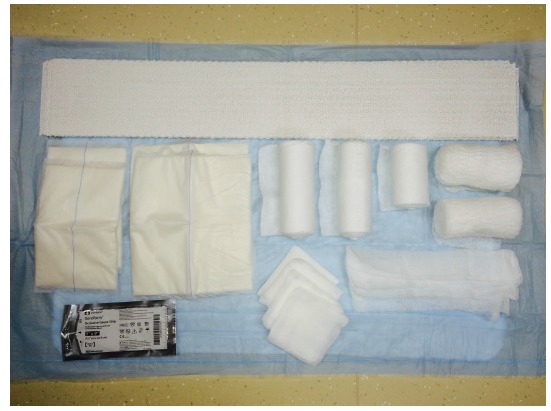
Materials routinely employed as described in the text for the modified Sir Robert Jones dressing. Note that a 6-inch double ACE wrap is missing.

A retrospective comparative study was performed with a chart review of 68 patients. All patients undergoing either primary or revision total ankle replacement between May 2011 and May 2015 were included. No patients were excluded. Four different types of prostheses were used: Salto Talaris Anatomical Ankle Prosthesis or Salto Talaris XT Revision Ankle Prosthesis (Integra Lifesciences, Plainsboro, NJ); Inbone II Total Ankle Replacement System (Wright Medical, Arlington, TN); and Agility Total Ankle System (DePuy Synthes, West Chester, PA) with a Revision, LP or custom-made long- stem LP talar component. All patients underwent a standardized surgical approach to total ankle replacement. All total ankle replacements were performed under general anesthesia and a single injection popliteal and saphenous nerve block with patient in the supine position and Foley catheter placed. Peri-operative antibiotics were administered between 30 and 60-minutes prior to initial incision, after 4-hours of open incision time (if encountered), and for 72-hours after the incision was closed. An indwelling closed suction drain was sutured in place intra-operatively and removed on post-operative day 3 or when the output was less than 1-cc/hr. The anterior incision itself was meticulously closed in layered fashion with absorbable braided sutures used for deep closure and a combination of nylon sutures and staples for closure of the skin. The patients were on strict bed rest until after the drain was removed and incision was inspected. At this point, they all participated in physical and occupational therapy to ensure their ability to safely keep all weight off of the operative extremity. Once the patients were able to demonstrate this, they were discharged either home or to a skilled nursing facility. They were instructed not to place any weight on the operative extremity until the prosthesis was securely bonded to the bone as demonstrated on weightbearing ankle radiographs obtained at 8-weeks post-operative or whenever the incision was deemed fully healed if beyond 8-weeks. The first 35 patients received a modified Sir Robert Jones type dressing (Fig. **2**). The dressing consisted of a non-adherent contact layer; 4-inch x 4-inch gauze; one 4-inch cotton under cast padding roll; two 6-inch cotton under cast padding rolls; two 9-inch x 5-inch ABD pads; two 8-inch x 10-inch ABD pads; and one 4 ½-inch gauze roll as previously described [12]. This was reinforced with a 15-thick, 5-inch by 30-inch posterior plaster splint mold and secured with an additional 4 ½-inch gauze roll and covered with a double 6-inch ACE wrap. The next 33 patients had the addition of an anterior offloading aperture pad made from cotton cast padding Fig. (**3**) applied directly over the surgical site then followed by the application of a modified Sir Robert Jones type dressing. The aperture pad consists of one 6-inch by 4-yard roll of cotton undercast padding unrolled back and forth on itself to an appropriate length. This typically is between 1/2 and 1/3 of a total roll. The pad is then manually split along its long axis and slightly spread open, thereby creating a central aperture of approximately the length of the anterior ankle incision.

The modified Sir Robert Jones dressing and splint are initially applied in the operating room in a sterile environment. The patients are then followed for weekly dressing changes. Once the sutures are removed, the patient is transitioned to a long leg removable boot with a mild tubular compression bandage worn underneath. In addition to the modified Sir Robert Jones dressing, a standard post-operative regimen for edema controlled is implemented. The patients were instructed not to place any weight on the operative foot and to spend 45-minutes of every waking hour with the operative leg above heart level until instructed otherwise. They were also instructed to apply ice to the popliteal fossa region of the operative knee for 15-minutes every waking hour until instructed otherwise. Deep venous thrombosis prophylaxis was employed using mechanical and pharmacological means until the patient was fully ambulatory at approximately 8-weeks post-operative.

Table **1** gives a comparison of patient demographics, type of prosthesis utilized, and known risk factors for incision wound healing between the two groups. There was no notable difference between the two groups. At each post-operative visit, the senior author performed the dressing change and noted any evidence of delayed incision healing, surgical site infections (SSI), pressure wounding, tendon tethering, neuropraxia or other complications. Incision wound healing problems were defined as the presence of incisional wound dehiscence, eschar or drainage after the index surgery but prior to the removal of sutures. SSI’s were defined according to criteria of the Centers for Disease Control and Prevention [13].

## 3 RESULTS

The dressings were tolerated by all the patients. Post-operatively, all patients returned to their next appointment with their dressing clean, dry and intact after each application. None required unplanned or emergent dressing changes. The outcome results are summarized in Table **2**. In the Control Group consisting of those in modified Sir Robert Jones compression dressings, there were 9 (25.7%) anterior incision wound healing problems. Of those, 1 (11.1%) went on to a below-knee amputation because of extensive plantar forefoot and anterior ankle soft-tissue necrosis precluding plastic surgery soft-tissue coverage further complicated by SSI and 1 (11.1%), although never clinically infected, required gracilis muscle free-tissue transfer for coverage of the anterior incision site because of delayed healing and resultant critical soft-tissue defect following débridement. The patient that underwent below-knee amputation suffered from type II diabetes, although well controlled, chronic kidney disease, and had a history of tobacco use. The possible cause of the dehiscence was secondary to pressure necrosis on the anterior incision. The patient requiring the free flap had no associated comorbidities or risk factors. The remaining 7 patients recovered with local wound cares with 2 requiring excision of the wound and layered closure in the office setting. None required a return to the operating room for closure. The Aperture Group had 3 (9.1%) incision wound healing problems. All of these resolved uneventfully using local wound cares with 1 requiring excision of the wound and layered closure in the office setting. None required a return to the operating room for closure. There were no SSI’s in the Aperture Group. Table **3** summarizes the co-morbidities. Although this data represents a difference of 16.6% in incision wound healing problems between the Control Group and the Aperture Group, using Pearson’s chi squared test, it was not found to be statistically significant (p=1.11). The numbers that would be needed to determine clinical significance would need to almost double the current enrollment or about 70 patients in each group. This problem was reflected in much of our findings. There was no significant difference in rates of additional incisions used during surgery between the Control Group and the Aperture Group (p=0.22). There was an improvement of greater than a week in time to incision healing (9.4 days) but this was also determined to not be statistically significant. Of note, the Aperture Group patients were significantly more likely to be undergoing a primary total ankle replacement (53.5% vs 28.6%, p = 0.042).

## 4 DISCUSSION

Delayed incision wound healing is a common post-operative complication that occurs with primary and revision total ankle replacement. For numerous factors, the anterior approach to surgical implantation poses its own risks to uncomplicated incision wound healing. In a 2012 study by Kessler *et al.* [14], they examined 26 deep periprosthetic infections involving total ankle replacements. Their results showed delayed incision wound healing does increase the chance of deep periprosthetic infection significantly (OR =15.38). With deep periprosthetic infection comes the inherent risk of prolonged treatment, additional surgeries, and even below-knee amputation. As reported by Myerson *et al.* [15] in 2014, once a deep periprosthetic infection develops following primary or revision total ankle replacement only a few will undergo successful joint-preserving revision total ankle replacement. The cost associated treatment of delayed incision wound healing and deep periprosthetic infection will vary, but Whalen *et al.* [8] estimated it would be approximately 5-times greater than if the primary total ankle replacement had healed uneventfully.

Given this increase in cost and the common occurrence of delayed incision wound healing, researches have looked at risk factors that might help predict and prevent delayed healing. Some authors have attempted to predict which patients would be more susceptible to incision wound healing problems. Studies have shown that a history of smoking tobacco ≥ 12-pack-years, peripheral vascular disease, and cardiovascular disease will increase the risk [2, 3]. A systematic review published in February of 2015 by Zhu *et al.* [16] reported a laundry list of significant factors including body mass index, diabetes mellitus, corticosteroid therapy and a history of rheumatoid arthritis. Screening for appropriate patients on the front end would hopefully reduce the incidence but appropriate post-operative dressings have their role as well.

Currently, there is no standard for post-operative dressing following primary or revision total ankle replacement. Researchers have examined different post-operative dressing techniques that may reduce the incidence of delayed wound healing by protecting the incision from post-operative edema [10, 11]. The data is scant but at least one study does show a significant decrease in incision wound healing problems with the use of NPWT [10]. The ideal dressing should be simple to apply, reproducible, and inexpensive. Prior studies involving dressings suffer from being labor intensive, expensive or both [10, 11].

The Sir Robert Jones compression dressing and its variants have been used for many years for both trauma and elective extremity surgeries. It originally consisted of layers of cotton wool, stockinette and elastic cloth applied in a tight but not constrictive fashion. The principle was to apply even pressure across the operative site and surrounding limb to reduce post-operative edema; however, not so much as to impair basic limb perfusion [17]. One of the authors had been using a modified version of the Sir Robert Jones dressing for use after primary and revision total ankle replacements to limit the naturally occurring edema. Recently, they added an additional modification which is present in this comparative study. This offloading device added to the modified Sir Robert Jones dressing is easy to create, simple to apply and cheap to produce. Fabricating the extra padding is as simple as creating a central tear in the layered cotton cast padding. The dressing adds no more complexity to the dressing application process than would the addition of another ABD pad to the anterior ankle. Our technique also is the least expensive seen in the literature. At our institution, the addition of the cotton cast padding adds approximately United States $2.19 to the cost of the dressing if 1 sterile 6-inch by 4-yard roll is used and only 99-cents if an unsterile same sized roll is used. We theorize that applying the aperture pad to the anterior ankle incision distributes the compressive forces created by the dressing and post-operative edema from the incision to the surrounding, intact tissue. We believe that this decrease in pressure allows for better tissue perfusion, reduced chance of pressure necrosis and overall reduction in delayed incision wound healing.

The data presented here does not demonstrate a statistically significant difference in incision wound healing complications between those whose dressing did or did not have the aperture applied. There would need to be a significant increase in the number of future patients with delayed incision wound healing to verify a statistically significant difference. The potential for a learning curve effect could exist because the prosthetic devices implanted during the study period changed, as did the frequency of primary and revision total ankle replacement. These may have reduced the soft-tissue dissection and manipulation to obtain prosthesis implantation, tourniquet time, and requirement for ancillary procedures to obtain a stable and balanced ankle. We did not have any way of controlling these potential factors and accordingly, the true effect, if any, a potential learning curve had on the reduction in anterior incision wound healing complications remains a matter for conjecture. In general, the use of statistical methods with small patient numbers implies the risk of a type II error. We believe it would be irresponsible to return to the original modified Sir Robert Jones Dressing for post-operative care of patients undergoing primary or revision total ankle replacement since there was a near 3-fold decrease in the number of patients with anterior incision wound healing complications when the cotton aperture pad was incorporated. This was enough to be considered clinically significant and prompted us to completely abandon the use of post-operative dressings without the aperture padding for primary and revision total ankle replacement. Due to the cost discrepancy between the techniques, future comparison of this anterior offloading aperture pad with single application disposable NPWT at 100-mmHg for both primary and revision total ankle replacement seems warranted.

## CONCLUSION

Delayed incision wound healing after primary and revision total ankle replacement is common. The cost of treating the natural sequelae of anterior incision wound healing problems can be high. We present a modification of the Sir Robert Jones dressing with addition of an anterior offloading aperture pad that is easy to manually fashion from one 6-inch by 4-yard cotton undercast padding roll. Our practice, although not statistically significant, this has reduced the number of patients with anterior incision wound healing problems 3-fold after primary and revision total ankle replacement. This technique is simple, reproducible and cost effective.

## Figures and Tables

**Fig. (3):**
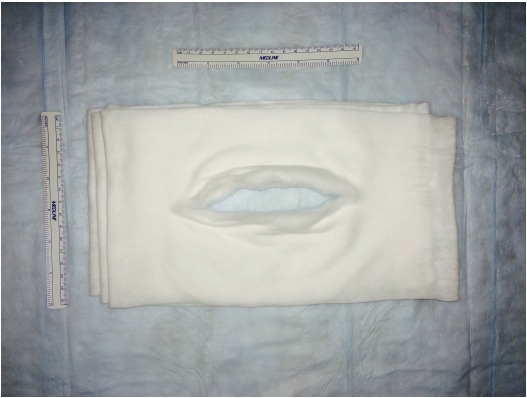
Photograph with rulers of the completed anterior offloading aperture pad made from one 6-inch by 4-yard roll of cotton undercast padding unrolled back and forth on itself and centrally split for the length of the anterior ankle incision.

**Table 1:** Demographics and co-morbidities of patients.

	**Control Group (N=35)**	**Aperture Group (N=33)**
**Mean age at surgery, years**	**63.1 ± 11.3**	**64.4 ± 10.6**
**Operative side**		
Right	**19**	**12**
center	**16**	**21**
**Gender**		
Male	**21**	**16**
Female	**14**	**17**
**Body Mass Index, kg/m^2^**	**31.1 ± 6.1**	**32.0 ± 5.6**
**Total ankle replacement type**		
Primary	**10**	**18**
Revision	**25**	**15**
**Comorbidities**		
Type II diabetes	**8**	**5**
Rheumatoid arthritis	**1**	**0**
Connective tissue disorder	**0**	**1**
Tobacco use	**4**	**6**
Immunosuppressive drugs	**1**	**0**
Chronic kidney disease	**3**	**5**
Coronary artery disease	**4**	**3**
**Type of prosthesis**		
AGILITY™ Revision/LP/Custom-made long-stemmed LP Talar Implant	**18**	**6**
INBONE™ II	**17**	**8**
SALTO TALARIS™	**0**	**10**
SALTO Talaris XT™	**0**	**9**
**Additional number of incisions**	**1.0 ± 0.7**	**1.1 ± 1.2**

**Table 2:** Summary of outcomes between the control group and the aperture group.

	**Control Group (N=35)**	**Aperture Group (N=33)**
**Incision wound healing problems**	**9 (25.7%)**	**3 (9.1%)**
Surgical site infections	**1 (2.9%)**	**0 (0%)**
**Total days from index surgery to complete suture removal**	**43.5 ±23.3**	**34.1 ± 13.0**
**Tendon tethering**	**10 (28.6%)**	**6 (18.2%)**
Resolved	**7 (70%)**	**0 (0%)**
**Neuropraxia/anesthesia**	**14 (40%)**	**5 (15.2%)**
Resolved	**3 (8.6%)**	**0 (0%)**

**Table 3:** Summary of co-morbidities in patients with incision wound healing complications.

	**Control Group (N=9)**	**Aperture Group (N=3)**
**Co-morbidities**		
Type II diabetes	**5 (55.6%)**	**2 (66.7%)**
Rheumatoid arthritis	**0 (0%)**	**0 (0%)**
Connective tissue disorder	**0 (0%)**	**0 (0%)**
Tobacco use within 1-year of surgery	**3 (33.3%)**	**2 (66.7%)**
Immunosuppressive drugs	**1 (11.1%)**	**0 (0%)**
Chronic kidney disease	**1 (11.1%)**	**1 (33.3%)**
Coronary artery disease	**2 (22.2%)**	**0 (0%)**
